# Special Issue “Evolution and Diversity of Insect Viruses”

**DOI:** 10.3390/v14010002

**Published:** 2021-12-21

**Authors:** Eugene V. Ryabov, Robert L. Harrison

**Affiliations:** 1 USDA, Agricultural Research Service, Bee Research Laboratory, Beltsville, MD 20705, USA; 2 USDA, Agricultural Research Service, Invasive Insect Biocontrol and Behavior Laboratory, Beltsville, MD 20705, USA

Insects are crucial for ecosystem functions and services and directly influence human well-being and health. Insects are among the most diverse classes of the animal kingdom, and therefore it is not surprising that they contain an immense degree of virus diversity, which remains largely unexplored. Until recently, investigations of insect virus diversity were focused on insect species of economic, veterinary, or medical importance. The deployment of high-throughput sequencing technologies has allowed for studies of virus diversity in a wider range of insect species and has led to an explosion in the discovery of new viral sequences in insect hosts, both from known virus groups and from entirely novel families. These studies have, in turn, inspired a re-assessment of the evolutionary relationships among, and taxonomic classification of, insect viruses. It is now clear that further studies of the biological effects of viruses on their insect hosts are required to understand the impact of diverse insect viromes on their host’s physiology, reproduction, and the potential of host insects to be viral vectors.

The aim of this Special Issue was to invite research articles focusing on recent advances in virus discovery and the elucidation of virus diversity in insect hosts. The published papers illustrated the effectiveness of high-throughput sequencing approaches for the comprehensive analysis of virus communities, identification of novel virus variants, and discovery of novel viruses. This includes the discovery of previously unknown RNA viruses in house crickets (*Acheta domesticus*) [1], soybean thrips (*Neohydatothrips variabilis*) [2], termites [3], dung flies (*Scathophaga furcata*) [4], mosquitoes (*Aedes aegypti*) from the Amazon basin [5], hematophagous arthropods (including mosquitoes, ticks, and bedbugs) from Serbia [6], honey bees (*Apis mellifera*) [7,8,9] and wild bee species (*Andrena* spp.) [7], and even *Varroa destructor* mites, which are closely associated with both Western and Eastern honey bees (*Apis mellifera* and *A. cerana*, respectively) [10]. Reports of the discovery and analysis of novel DNA viruses, including nudiviruses of corn rootworms (*Diabrotica undecimpunctata* and *D. virgifera*) [11] and a betabaculovirus from the moth *Matsumuraeses phaseoli*, an important legume pest, were also presented [12].

Sequence data on the novel viruses identified in high-throughput sequencing studies could be a starting point for further analysis of virus–host interactions. Notably, some of the published papers already present such analyses, including the investigation of viral inter-species transmission between wild and managed honey bees [7] and between the mites *V. destructor* and honey bees (*A. mellifera*) [10], and analysis of virus-derived small interfering RNAs providing an insight into antiviral RNA responses [4].
